# Using Two-dimensional Principal Component Analysis and Rotation Forest for Prediction of Protein-Protein Interactions

**DOI:** 10.1038/s41598-018-30694-1

**Published:** 2018-08-27

**Authors:** Lei Wang, Zhu-Hong You, Xin Yan, Shi-Xiong Xia, Feng Liu, Li-Ping Li, Wei Zhang, Yong Zhou

**Affiliations:** 1 College of Information Science and Engineering, Zaozhuang University, Zaozhuang, 277100 P.R. China; 2 Xinjiang Technical Institute of Physics and Chemistry, Chinese Academy of Science, Urumqi, 830011 P.R. China; 3 School of Computer Science and Technology, China University of Mining and Technology, Xuzhou, 221116 P.R. China; 4 School of Foreign Languages, Zaozhuang University, Zaozhuang, 277100 P.R. China; 5 China National Coal Association, Beijing, 100713 P.R. China

## Abstract

The interaction among proteins is essential in all life activities, and it is the basis of all the metabolic activities of the cells. By studying the protein-protein interactions (PPIs), people can better interpret the function of protein, decoding the phenomenon of life, especially in the design of new drugs with great practical value. Although many high-throughput techniques have been devised for large-scale detection of PPIs, these methods are still expensive and time-consuming. For this reason, there is a much-needed to develop computational methods for predicting PPIs at the entire proteome scale. In this article, we propose a new approach to predict PPIs using Rotation Forest (RF) classifier combine with matrix-based protein sequence. We apply the Position-Specific Scoring Matrix (PSSM), which contains biological evolution information, to represent protein sequences and extract the features through the two-dimensional Principal Component Analysis (2DPCA) algorithm. The descriptors are then sending to the rotation forest classifier for classification. We obtained 97.43% prediction accuracy with 94.92% sensitivity at the precision of 99.93% when the proposed method was applied to the PPIs data of *yeast*. To evaluate the performance of the proposed method, we compared it with other methods in the same dataset, and validate it on an *independent* datasets. The results obtained show that the proposed method is an appropriate and promising method for predicting PPIs.

## Introduction

Since the interactions among proteins play an extremely important role in almost all biological processes, many researchers have designed innovative techniques for detecting Protein-Protein Interactions (PPIs) in post genome era^1,2^. Over the past several decades, various high-throughput techniques have been proposed and designed, including yeast two-hybrid (Y2H) system^2–4^, microarray analysis^5^, and mass spectrometry^4,6^, for large-scale and systematic prediction of PPIs. However, the PPIs determined from these traditional biological experiment methods only accounts for a small proportion of the PPIs network^7–9^. In addition, the high-throughput experiment methods are usually expensive and time-consuming with high ratio of both false-positives and false-negatives^10–13^. To predict the PPIs more efficiently and at low cost, various computational-based approaches have been proposed so far to solve this problem^14–19^. These computational approaches can be roughly classified into structure based methods, literature knowledge based methods, network topology based methods, and genome based methods, according to the information they perform on their tasks^20^. However, the application of these approaches is restricted, because they can hardly be practiced if the pre-knowledge of the proteins is unavailable^19,21,22^.

More recently, researchers have become increasingly interested in determining whether proteins interact by using information obtained directly from the protein amino acid sequence^13,23–26^. Numerous studies have indicated that the information extracted from protein amino acid sequences alone is sufficient to predict the interactions of proteins^27,28^. Pitre *et al*. proposed the PIPE algorithm based on the hypothesis that some of the protein interactions are mediated by a limited number of short polypeptide sequences. In the detection of yeast protein interactions PIPE realized an overall accuracy of 75% with 61% sensitivity and a specificity of 89%^29^. Shen *et al*. using only protein sequences information developed a method for PPIs prediction. The method combines the conjoint triad feature for describing amino acids and learning algorithm based on support vector machine (SVM). In the five-fold cross-validation, they achieved an accuracy of 83.90%^30^. Guo *et al*. combined the automatic covariance features extracted from the protein amino acid sequences and the support vector machine classifier to predict the interaction among proteins. This method has obtained an average accuracy of 86.55% when performed on the *Saccharomyces cerevisiae* dataset^7^.

In this article, we develop a new sequence-based approach to predict PPIs using the matrix-based protein sequence descriptors combined with the Rotation Forest (RF). In detail, we first represent the protein sequence as the Position-Specific Scoring Matrix (PSSM) and use the two-dimensional Principal Component Analysis (2DPCA) algorithm to extract numerically descriptor to characterize the protein amino acid sequence. We then construct the feature vector of the protein pair by coding two protein vectors in this pair. Finally, the feature vectors of these protein pairs are sent to the RF classifier for classification. In order to assess the ability of the proposed model to predict PPIs, we use *Yeast* and *Helicobacter pylori* datasets to verify it. In the experiment, our model achieved 97.43% and 88.07% prediction accuracy with 94.92% and 78.20% sensitivity at the specificity of 99.93% and 97.44% on these two datasets. Furthermore, we evaluated the ability of the proposed model on *independent* datasets (*C*.*elegans*, *E*.*coli*, *H*.*sapiens* and *M*.*musculus*), where 91.43%, 99.93%, 92.00% and 90.73% of the prediction accuracy were generated, respectively.

## Results and Discussions

### Evaluation Criteria

In this study, we use five-fold cross-validation technique to verify the predictive power of our model. All samples are randomly divided into almost the same number of 5 subsets, each subset containing interacting and non-interacting protein pairs. Four subsets are used as training sets each time, and the remaining one subset is used as a test set, the process is repeated five times so that every subset is used as a test set once. The performance of the method is the average of the 5 sets performances. Several evaluation criteria used in our study to estimate the predictive power of our model including accuracy (Accu.), sensitivity (Sen.), precision (Prec.), and Matthews correlation coefficient (MCC). The calculation formulas are listed below:

1 $$Accu.=\frac{TP+TN}{TP+TN+FP+FN}$$

2 $$Sen.=\frac{TP}{TP+FN}$$

3 $$Prec.=\frac{TP}{TP+FP}$$

4 $$MCC=\frac{TP\times TN-FP\times FN}{\sqrt{(TP+FP)(TP+FN)(TN+FP)(TN+FN)}}$$

where True Positive (TP) represents the number of correct classification of positive samples, False Positive (FP) represents the number of incorrect classification of positive samples, True Negative (TN) represents the number of correct classification of negative samples, and False Negative (FN) represents the number of incorrect classification of negative samples.

We also produce Receiver Operating Characteristic (ROC) curves^31^ to estimate the performance of the classifier. Typically, the random classification threshold of the two-class classifier is 0.5. When the new classification results are accepted, the threshold will change along with the true positive rate and the false positive rate, and this change will be plotted in the form of graphics. In addition, the Area Under a Curve (AUC) is calculated in the experiment. The performance of different prediction methods can be expressed directly with AUC values, which is considered to be better than the other method when the AUC value of one method is greater than the value of another method.

### Evaluation of model predictive ability

We appraise the ability of our model using the Golden Standard Datasets. To ensure the stability of the experimental results, the five-fold cross-validation is exploited in the experiment. The parameters of the rotation forest (feature subset number K and decision trees number L) were tested within the range of values by the grid search method to expect to achieve better performance. Considering the accuracy rate and time cost of the rotation forest, as a result the best parameter we get *K* is 20 and *L* is 2.

**Figure 1:**
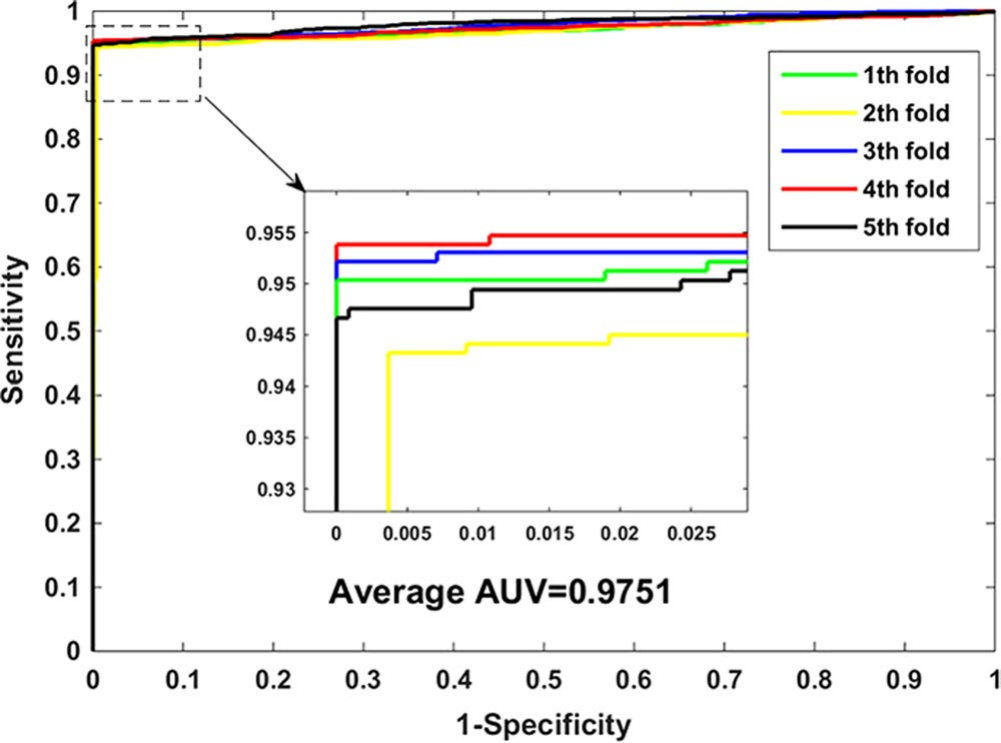
The ROC curves performed on the *Yeast* dataset using the proposed method.

The experimental results of the RF classifier and the matrix-based protein amino acids sequences representation is summarized in Table 1. As seen from the Table 1 that the average accuracy of our approach is as high as 97.43%. In order to more fully show the predicted results of our approach, we also calculated the values of precision, sensitivity, MCC, and AUC. From Table 1, we can see that our model has achieved good experimental results, the sensitivity value of 94.92%, the precision value of 99.93%, the MCC value of 94.97%, and the AUC value of 97.51%. Furthermore, it can be seen from the table that the standard deviation of accuracy, sensitivity, precision, MCC, and AUC is 0.30%, 0.43%, 0.17%, 0.59% and 0.47%, respectively. Figure 1 plots the ROC curve generated by our method on the *Yeast* dataset. X-axis expresses false positive rate (FPR) and Y-axis expresses true positive rate (TPR) in the figure.

**Table 1:** The five-fold cross-validation results achieved on the *Yeast* dataset using the proposed method.

Testing set	Accu. (%)	Sen. (%)	Prec. (%)	MCC (%)	AUC (%)
1	97.50	95.04	100.00	95.11	97.27
2	96.92	94.32	99.63	93.98	96.88
3	97.63	95.22	100.00	95.37	97.89
4	97.68	95.38	100.00	95.46	97.46
5	97.41	94.66	100.00	94.94	98.05
**Average**	**97**.**43** ± **0**.**30**	**94**.**92** ± **0**.**43**	**99**.**93** ± **0**.**17**	**94**.**97** ± **0**.**59**	**97**.**51** ± **0**.**47**

**Figure 2:**
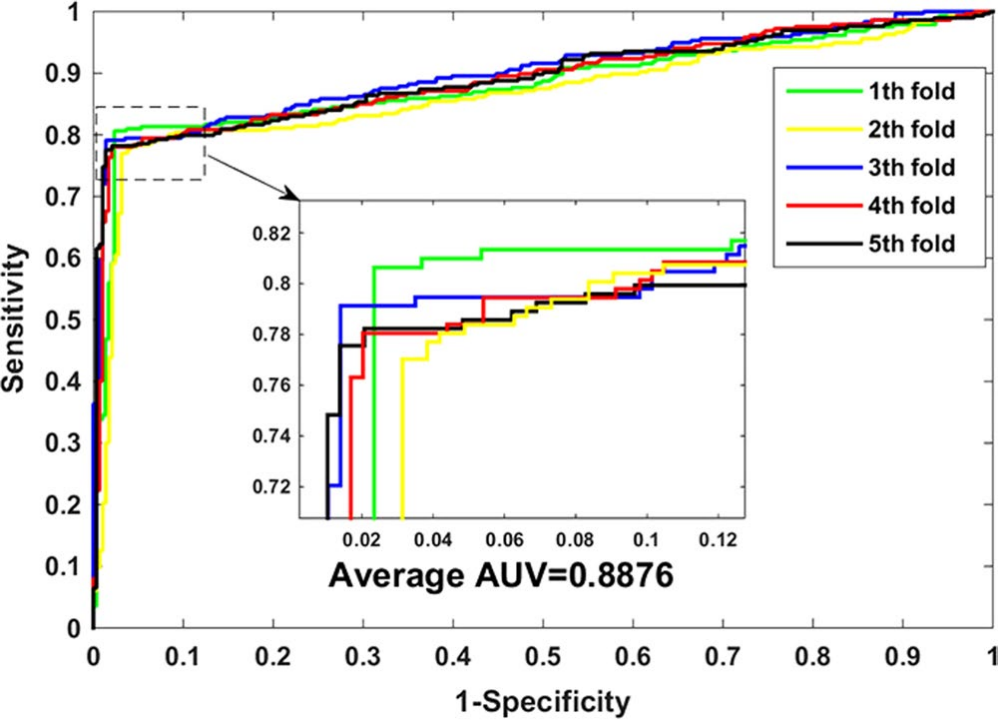
The ROC curves performed on the *H*. *pylori* dataset using the proposed method.

In order to further evaluate the ability of our approach to predict PPIs, we tested it against the *H*. *pylori* dataset. In the experiment, the same classifier parameters and feature extraction algorithm are used. Table 2 lists the experimental results of cross-validation. We achieved the high accuracy of 88.07%, the sensitivity value of 78.20%, the precision value of 97.44%, the MCC value of 77.66%, and the AUC value of 88.76% on the *H*. *pylori* dataset. In addition, from Table 2 we can also observe that the standard deviation of accuracy, sensitivity, precision, MCC, and AUC is 0.77%, 0.97%, 0.90%, 1.46% and 1.32%, respectively. Figure 2 plots the ROC curve generated by our method on the *H*. *pylori* dataset.

**Table 2:** The five-fold cross-validation results achieved on the *H*. *pylori* dataset using the proposed method.

Testing set	Accu. (%)	Sen. (%)	Prec. (%)	MCC (%)	AUC (%)
1	88.68	79.23	96.98	78.51	88.36
2	86.79	77.03	96.20	75.21	86.71
3	88.68	79.12	98.33	79.00	90.22
4	88.16	78.05	97.39	77.76	89.18
5	88.01	77.55	98.28	77.83	89.33
**Average**	**88**.**07** ± **0**.**77**	**78**.**20** ± **0**.**97**	**97**.**44** ± **0**.**90**	**77**.**66** ± **1**.**46**	**88**.**76** ± **1**.**32**

### Comparison of the proposed model with different classifiers and descriptors

Machine learning has been successfully and reliably applied to predictive PPIs. Wherein, SVM is one of the famous algorithms based on statistical learning theory. To verify the predictive ability of our approach, we compare the RF classifier with the SVM classifier based on the same feature extraction method. For the SVM classifier, the LIBSVM we used can be downloaded at www.csie.ntu.edu.tw/~cjlin/libsvm, which was originally proposed by Chang and Lin^32^. The grid search method is used to optimize SVM parameters and the optimal parameters c and g on this dataset are 0.1 and 0.5, respectively.

**Figure 3:**
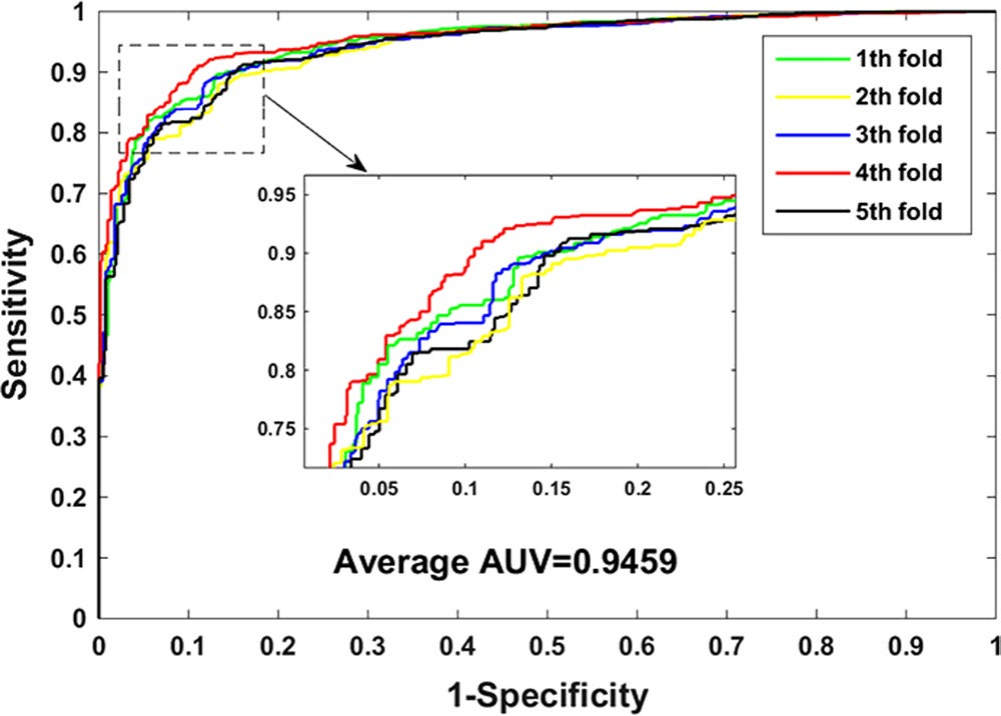
The ROC curves performed on the *Yeast* dataset using the SVM classifier.

The experimental prediction results of the SVM combined with the protein sequence descriptor are listed in Table 3. It can be observed from Table 3 that the accuracy of SVM on the *Yeast* dataset is 87.29%, wherein the results of five experiments are 87.84%, 85.47%, 87.71%, 89.23%, and 86.21%. However, the rotation forest classifier achieves an average accuracy of 97.43%. To show the prediction ability of our approach more comprehensively, we calculated the values of precision, sensitivity, MCC, and AUC. As seen from the Table 3, the prediction result of the SVM classifier with the sensitivity value of 84.42%, precision value of 89.58%, MCC value of 74.73%, and AUC value of 94.59%. Furthermore, we can see in detail from Table 3 that the standard deviation of accuracy, sensitivity, precision, MCC, and AUC is 1.48%, 1.88%, 2.02%, 2.93% and 0.56%, respectively. The accuracy, sensitivity, precision, MCC and AUC of the RF classifier is 10.14%, 10.50%, 10.35%, 20.24% and 2.92% higher than that of the SVM classifier. From the comparison of experimental results we can see that the evaluation criteria based on SVM classifier are all lower than those of our model. The ROC curves performed by support vector machine classifier on *Yeast* dataset were shown in Fig. 3.

**Table 3:** The five-fold cross-validation results achieved on the *Yeast* dataset using the SVM classifier.

Testing set	Accu. (%)	Sen. (%)	Prec. (%)	MCC (%)	AUC (%)
1	87.84	85.37	90.00	75.79	94.80
2	85.47	81.48	89.20	71.27	94.13
3	87.71	83.84	90.63	75.60	94.41
4	89.23	86.41	91.71	78.59	95.47
5	86.21	85.00	86.36	72.38	94.16
Average	87.29 ± 1.48	84.42 ± 1.88	89.58 ± 2.02	74.73 ± 2.93	94.59 ± 0.56
**Our method**	**97**.**43** ± **0**.**30**	**94**.**92** ± **0**.**43**	**99**.**93** ± **0**.**17**	**94**.**97** ± **0**.**59**	**97**.**51** ± **0**.**47**

To further evaluate the performance of our approach, we also compared it with different descriptors. In the experiment, we selected feature extraction algorithms including Auto Covariance (AC) and Discrete Cosine Transform (DCT) to perform experiments on the *Yeast* dataset. The introduction of these feature extraction algorithms can be viewed in the supplementary file.In addition, we also verified the protein descriptors without feature extraction. Table 4 summarizes the comparison results of the proposed feature descriptor with the above three descriptors. It can be seen from Table 4 that our feature descriptors have obtained the best results on accuracy, sensitivity, and MCC. The precision is only 0.07% lower than the highest AC descriptor and DCT descriptor. This indicates that the 2DPCA algorithm can effectively extract the features of the protein and help improves the prediction performance of the model.

**Table 4:** The performance comparison among different descriptor on the *Yeast* dataset.

Descriptor	Accu. (%)	Sen. (%)	Prec. (%)	MCC (%)
AC	93.14 ± 0.69	86.28 ± 1.23	**100**.**00** ± **0**.**00**	87.10 ± 1.20
DCT	93.65 ± 0.67	87.30 ± 1.41	**100**.**00** ± **0**.**00**	88.02 ± 1.21
Original	81.50 ± 0.62	70.55 ± 0.51	90.33 ± 1.94	64.57 ± 1.65
2DPCA	**97**.**43** ± **0**.**30**	**94**.**92** ± **0**.**43**	99.93 ± 0.17	**94**.**97** ± **0**.**59**

### Comparison with Existing Method

In the past few years, many research teams have put forward a variety of computational methods to solve the problem of PPI prediction. By comparison with these models on the *Yeast* and *H*. *pylori* datasets, we can more clearly evaluate the proposed method. We selected accuracy, precision, sensitivity, and MCC as evaluation indicators that are listed in Tables 5 and 6. Table 5 summarizes the experimental results of different approaches on the *Yeast* dataset. From the table we can clearly see that the range of accuracy generated by the other methods is from 75.08% to 89.33%, the range of sensitivity generated is from 75.81% to 89.93%, the range of precision generated is from 74.75% to 90.24%, and corresponding experimental results we generated were 97.43%, 94.92%, 99.93%, 94.97%, these results are lower than what we have achieved. Table 6 shows the performance of different models on the *H*. *pylori* dataset. It can be seen that the range of accuracy generated by the other approaches is from 75.80% to 87.50%, the range of sensitivity obtained is from 69.80% to 88.95%, the range of precision obtained is from 80.20% to 86.15%, and the corresponding experimental results we obtained were 88.07%, 78.20%, 97.44%, and 77.66%. Except for the precision and MCC slightly lower, the accuracy and sensitivity are higher than the highest value.

**Table 5:** The performance comparison between different methods on the *Yeast* dataset.

Author	Model	Accu. (%)	Sen. (%)	Prec. (%)	MCC (%)
Guos’ work^7^	ACC	89.33 ± 2.67	89.93 ± 3.68	88.87 ± 6.16	N/A
	AC	87.36 ± 1.38	87.30 ± 4.68	87.82 ± 4.33	N/A
Zhous’ work^40^	SVM + LD	88.56 ± 0.33	87.37 ± 0.22	89.50 ± 0.60	77.15 ± 0.68
Yangs’ work^41^	Cod1	75.08 ± 1.13	75.81 ± 1.20	74.75 ± 1.23	N/A
	Cod2	80.04 ± 1.06	76.77 ± 0.69	82.17 ± 1.35	N/A
	Cod3	80.41 ± 0.47	78.14 ± 0.90	81.86 ± 0.99	N/A
	Cod4	86.15 ± 1.17	81.03 ± 1.74	90.24 ± 0.45	N/A
Yous’ work^42^	PCA-EELM	87.00 ± 0.29	86.15 ± 0.43	87.59 ± 0.32	77.36 ± 0.44
**Our method**	**RF** + **PSSM**	**97**.**43** ± **0**.**30**	**94**.**92** ± **0**.**43**	**99**.**93** ± **0**.**17**	**94**.**97** ± **0**.**59**

**Table 6:** The performance comparison of different methods on the *H*. *pylori* dataset.

Model	Accu. (%)	Sen. (%)	Prec. (%)	MCC (%)
Signature products^34^	83.40	79.90	85.70	N/A
Ensemble ELM^42^	87.50	88.95	86.15	**78.13**
Phylogentic bootstrap^43^	75.80	69.80	80.20	N/A
HKNN^44^	84.00	86.00	84.00	N/A
Ensemble of HKNN^45^	86.60	86.70	85.00	N/A
Boosting^46^	79.52	80.37	81.69	70.64
**Our method**	**88**.**07**	**78**.**20**	**97**.**44**	77.66

### Prediction Ability on Independent Datasets

To further estimate the proposed model, we decided to verify its performance on an *independent* datasets. We apply all of the 11188 pairs from the *Yeast* dataset as the training set in our final prediction model, the test set is composed of *C*.*elegans*, *E*.*coli*, *H*.*sapiens and M*.*musculus* datasets from the DIP database. The number of protein pairs they contained was 4013, 6954, 1413, and 313, respectively. In the experiment, we utilize the same matrix representation and feature extraction algorithm for these datasets, and we also use the same parameters for rotation forest classification. Table 7 lists the prediction results of four *independent* datasets based on our method. We can observe from Table 7 that the high accuracy of 91.43% was acquired on the *C*.*elegans* dataset, 99.93% accuracy on the *E*.*coli* dataset, 92.00% accuracy on the *H*.*sapiens* dataset, and 90.73% accuracy on the *M*.*musculus* dataset. All of these results demonstrate that our approach is a suitable method for predicting the interactions of other species.

**Table 7:** Predictive results of four species based on the proposed method.

Species	Test pairs	Accu. (%)
*C*.*elegans*	4013	91.43
*E*.*coli*	6954	99.93
*H*.*sapiens*	1412	92.00
*M*.*musculus*	313	90.73

## Conclusions

In this article, we develop an efficient and practical prediction approach, which utilizes protein sequence information combined with feature descriptors to accurately predict protein interactions at high speed. It is well known that the most important challenge of sequence-based algorithm is to find appropriate features to adequately represent the information of protein interactions. For this purpose, we transform the protein sequences into the PSSM and use the 2DPCA algorithm to extract their features, extracting as much as possible the hidden information in the primary sequence of the protein. Then the rotation forest is applied to guarantee the reliability of prediction. In comparison with the SVM classifier and other approaches, our model has achieved excellent results. Furthermore, we validate our model on the *independent* datasets. The excellent results show that our model performed well in the prediction of protein interactions. In future research, we will focus on finding better ways to describe protein sequences to accurately identify interacting and non-interacting protein pairs.

## Materials and Methodology

### Golden Standard Datasets

In the experiments we used the real *Yeast* PPIs dataset, which was collected from *Saccharomyces cerevisiae* core subset of Database of Interacting Proteins (DIP)^33^ by Guo *et al*.^7^. A total of 5966 interaction protein pairs are included in the Saccharomyces cerevisiae core subset. In order to remove the redundant in the dataset, we deleted protein pairs with the sequence identity of more than 40% or protein pairs with the protein residue of less than 50. The number of these redundant protein pairs is 372. Therefore, the remaining 5594 protein pairs constitute the positive dataset of golden standard. To construct the negative dataset, we in accordance with the hypothesis that the proteins do not interact with each other in different subcellular compartments, and strictly according to Guo’s work procedure, we finally obtained 5594 protein pairs. Therefore, the complete *Yeast* PPIs dataset contains 11188 pairs, half of which are from the positive dataset and half from the negative dataset. Another dataset we used was the *Helicobacter pylori* dataset collected by Martin *et al*.^34^ consisting of 2916 pairs. There are interacting protein pairs and non-interacting protein pairs each accounted for fifty percent.

### Position-Specific Scoring Matrix

Position-Specific Scoring Matrix (PSSM) is proposed by Gribskov *et al*.^35^ to detect distantly related protein. The structure of PSSM is a matrix of *N* rows and 20 columns. Suppose $M=\{{\varepsilon }_{i,j}:i=1\cdots N\,and\,j=1\cdots 20\}$ and each matrix is represented as follows:

5 $$M=[\begin{array}{cccc}{\varepsilon }_{1,1} & {\varepsilon }_{1,2} & \cdots  & {\varepsilon }_{1,20}\\ {\varepsilon }_{2,1} & {\varepsilon }_{2,2} & \cdots  & {\varepsilon }_{2,20}\\ \vdots  & \vdots  & \vdots  & \vdots \\ {\varepsilon }_{N,1} & {\varepsilon }_{N,2} & \cdots  & {\varepsilon }_{N,20}\end{array}]$$

where *ε*_*i*,*j*_ in the *i* row of PSSM mean that the probability of the *ith* residue being mutated into type *j* of 20 native amino acids during the procession of evolutionary in the protein from multiple sequence alignments.

In our experiment, we introduced the Position-Specific Iterated BLAST (PSI-BLAST) tool^26,36^ and the *SwissProt* database to create the PSSM for each protein amino acid sequence. The PSI-BLAST is a highly sensitive protein sequence alignment program that is effective in discovering new members of protein family and similar proteins in distantly related species. To obtain more homologous sequences, we set the e-value to 0.001, the number of iterations to 3, and the default value of the other parameters. We can download the *SwissProt* database and PSI-BLAST tool from, http://blast.ncbi.nlm.nih.gov/Blast.cgi.

### Two-dimensional Principal Component Analysis

Two-dimensional Principal Component Analysis (2DPCA)^37,38^ is an effective feature extraction algorithm based on two-dimensional matrix, which has been universally used in a variety of fields. It can be directly applied to the two-dimensional matrix and significantly reduces the computational complexity and the probability of singularity in feature extraction. The 2DPCA does not need to convert the matrix into a row vector or a column vector first, but directly uses the two-dimensional projection method for feature extraction. Studies have shown that extracting features directly from the matrix mode without vectorization preprocessing can not only reduce the computational complexity, but also improve performance in subsequent classification based on nearest neighbor rules. Therefore, the 2DPCA algorithm has the advantages of feature extraction directly, less generated feature data and less time consuming. The 2DPCA algorithm is described as follows.

Assuming that the sample number is *N* and the *ith* matrix is *V*_*i*_ (*i* = 1, 2, …, *N*), this means $\bar{V}$ can be calculated as follows:

6 $$\bar{V}=\frac{1}{N}\sum _{i=1}^{N}\,{V}_{i}$$

In the 2DPCA algorithm, the matrix *V* is projected onto the optimal projection matrix, so we can get the following formula:

7 $$F=VX$$

Thus we can get an M-dimensional projection vector *F*. The optimal projection axis *X* is determined by the dispersion of eigenvector *F*, and uses the following equation:

8 $$J(X)=trace({S}_{x})$$

where *S*_*x*_ denotes the covariance matrix of the projection eigenvector *F*, and *trace* (*S*_*x*_) denotes the trace of *S*_*x*_. The purpose of the maximizing criterion is to search for an optimal projection direction to maximize the total scatter matrix of the training samples. The covariance matrix *S*_*x*_ is represented as:

9 $$\begin{array}{c}{S}_{x}=E[F-E(F)]{[F-E(F)]}^{T}\\ \,=E[VX-E(VX)]{[VX-E(VX)]}^{T}\\ \,=E\{[V-E(V)]X\}{\{[V-E(V)]X\}}^{T}\end{array}$$

so,

10 $$trace({S}_{x})=trace({X}^{T}\{E{[V-E(V)]}^{T}[V-E(V)]\}X)$$

Define total scatter matrix *G*_*t*_ as:

11 $${G}_{t}=E\{{[V-E(V)]}^{T}[V-E(V)]\}$$

The formula for calculating *G*_*t*_ is:

12 $${G}_{t}=\frac{1}{N}\sum _{i=1}^{N}\,{({V}_{j}-\bar{V})}^{T}[V-E(V)]$$

Therefore, the criterion function can be written as:

13 $$J(X)=trace({X}^{T}{G}_{t}X)$$

where *X* is a unit column vector. The first *d* maximum eigenvalues of the covariance matrix corresponding to the orthogonal eigenvectors constitute the optimal projection axis *X*_1_, *X*_2_, …, *X*_*d*_. The matrix *V* is projected onto the vector *X*_1_, *X*_2_, …, *X*_*d*_, and extract its features, let

14 $${F}_{k}={\rm{V}}{X}_{k},\,k=1,2,\ldots ,d$$

A new set of eigenvectors *F*_1_, *F*_2_, …, *F*_*d*_, can be obtained by calculation (14), which is the principal component of matrix V. In the 2DPCA algorithm, we expect to find the appropriate number of projection axes so that it can reduce the dimensionality of the data without losing useful information.

### Rotation Forest

Rotation forest^16^ is a popular ensemble classifier which has been proposed recently. RF first divides the attributes set of samples randomly, and transforms the attribute subsets by means of linear transformation to increase the difference between the subsets. Then use the transformed attribute subsets to train different classifiers and finally obtain reliable classification results^39^.

Assume that {*x*_*i*_, *y*_*i*_} contains *T* samples, of which *x*_*i*_ = (*x*_*i*1_, *x*_*i*2_, …, *x*_*in*_) is an *n*-dimensional feature vector. Let *X* be the training sample set, *Y* be the label set and *S* be the feature set. *X* is a training set containing *T* training samples, forming a matrix of *T* × *n*. Suppose the number of decision trees is *D*, then the decision trees can be represented as *F*_1_, *F*_2_, …, *F*_*d*_. The rotation forest algorithm is implemented as follows.

1. Select the suitable parameter *K*, randomly divide *S* into *K* parts of the disjoint subsets, the number of features that each subset contains is $n/k$.
2. Let *S*_*i*,*j*_ be the *j-th* feature subset and use it for classifier *F*_*i*_ training. For each feature subset, a non-empty subset is randomly selected and repeatedly sampled in a certain proportion, forming a sample subset ${X^{\prime} }_{i,j}$.
3. Principal component analysis is performed on ${X^{\prime} }_{i,j}$ to obtain *M*_*i*,*j*_ principal components.
4. The coefficients obtained in the matrix *M*_*i*,*j*_ are constructed a sparse rotation matrix *G*_*i*_, which is expressed as follows:

15 $${G}_{i}=[\begin{array}{cccc}{a}_{i,1}^{(1)},\ldots ,{a}_{i,1}^{({D}_{1})} & 0\, & \cdots  & 0\\ 0 & {a}_{i,2}^{(1)},\cdots ,{a}_{i,2}^{({D}_{2})} & \cdots  & 0\\ \vdots  & \vdots  & \ddots  & \vdots \\ 0 & 0 & \cdots  & {a}_{i,k}^{(1)},\ldots ,{a}_{i,k}^{({D}_{k})}\end{array}]$$

During the prediction period, a test sample *x* generated by the classifier *F*_*i*_ of ${d}_{i,j}(X{G}_{i}^{a})$ is provided to determine that *x* belongs to class *y*_*i*_. Next, the class of confidence is calculated by means of the average combination, and the formula is as follows:

16 $${\lambda }_{j}(x)=\frac{1}{D}\sum _{i=1}^{D}\,{d}_{i,j}(X{G}_{i}^{a})$$

**Figure 4:**
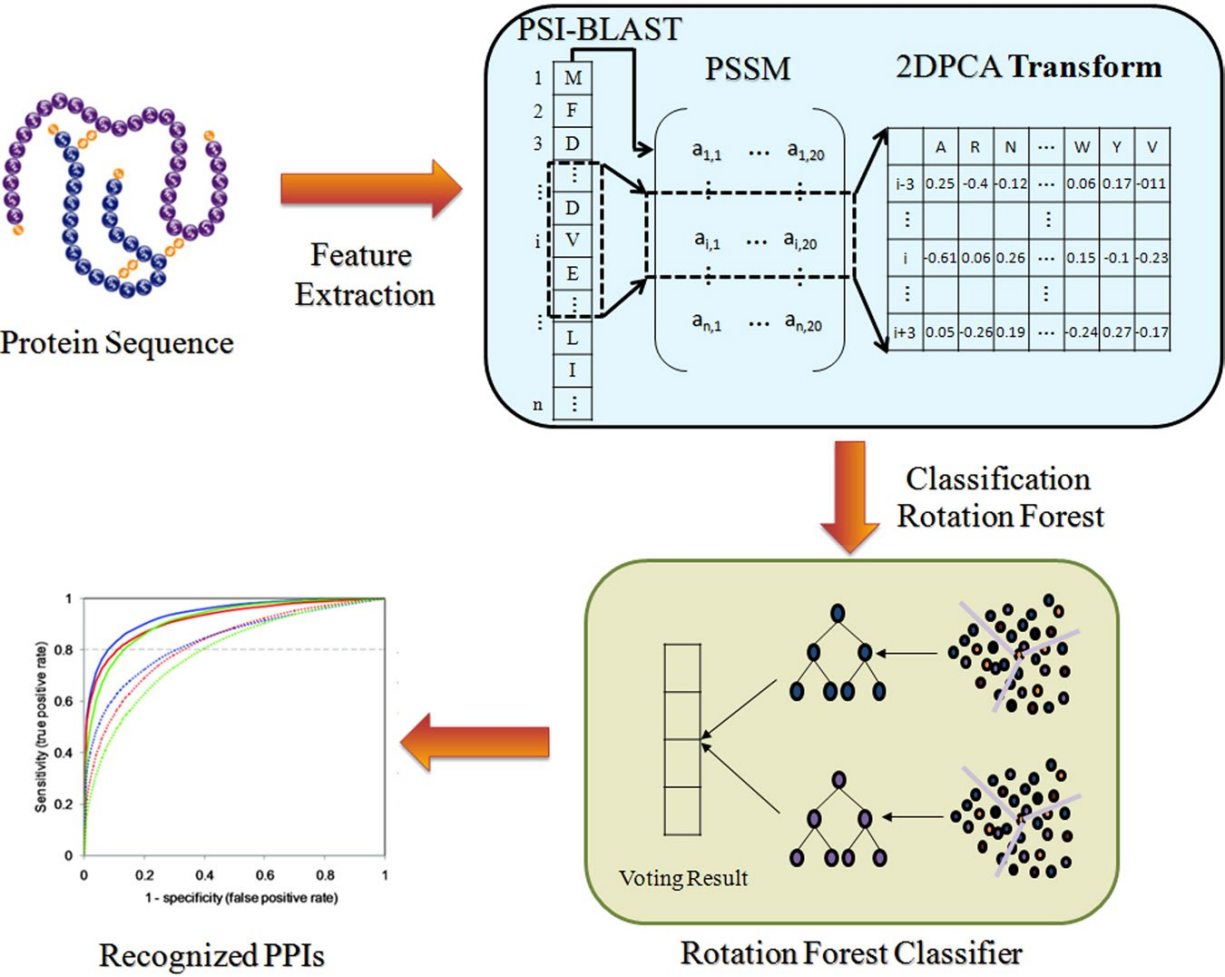
Flow chart of the proposed method.

Then assign the category with the largest *λ*_*j*_(*x*) value to *x*. The flow chart of our approach is shown as Fig. 4.

## Electronic supplementary material

Supplementary Materials
