# Supplementary material for: Using Two-dimensional Principal Component Analysis and Rotation Forest for Prediction of Protein-Protein Interactions
Source: Sci Rep. 2018 Aug 27;8:12874. doi: 10.1038/s41598-018-30694-1 (PMC6110764; doi:10.1038/s41598-018-30694-1)
Supplement: Supplementary file 1 — Supplementary Materials [file 41598_2018_30694_MOESM1_ESM.doc]

## **Supplementary Material**

| **Paper title:** | Using Two-dimensional Principal Component Analysis and Rotation Forest for Prediction of Protein-Protein Interactions |
| --- | --- |
| **Authors:** | Lei Wang, Zhu-Hong You, Xin Yan, Shi-Xiong Xia, Feng Liu, Li-Ping Li, Wei Zhang, Yong Zhou |

This Supplementary Material consists of a brief description of the *feature representation methods and performance measures.*

**Feature representation methods**

**Conjoint triad (CT)**

Conjoint triad (CT) (Shen et al. 2007) considers the properties of one amino acid and its vicinal amino acids and regards any three continuous amino acids as a unit. Thus, the triad can be differentiated according to the classes of amino acid. The PPI information of protein sequence can be projected into a homogeneous vector space by counting the frequency of each triad type. It should be noted that before using such feature representation method, the 20 amino acids has been clustered into seven classes according to the dipoles and volumes of the side chains. The classification of amino acids is listed in Supplementary Table S1. And thus the dimensions of a protein sequence were dramatically reduced to 7×7×7 =343. Finally, the descriptors of two proteins were concatenated and a total 686-dimensional vector has been built to represent each protein pair. For detailed descriptions of these autocorrelation descriptors, interested readers could refer to (Shen et al. 2007).

**Auto covariance (AC)**

Given a protein sequence, auto covariance (AC) (Guo et al. 2008) accounts for the interactions between amino acids with a certain number of amino acids apart in the sequence, so this method takes neighboring effect into account and makes it possible to discover patterns that run through entire sequences. By this means, the amino acid residues were first translated into numerical values representing physicochemical properties. Here six sequence-based physicochemical properties of amino acids based on our previous studies (Shi et al. 2009; Xia et al. 2010; Yang et al. 2010) were chosen to reflect the amino acids characteristics. Table S2 in Supplementary showed the values of the six physicochemical properties for each amino acid. And then AC was used to transform these numerical sequences into uniform matrices.

**Local descriptor (LD)**

Local descriptor (LD) (Yang et al. 2010) is an alignment-free approach and its effectiveness depends largely on the underlying amino acid groups. To reduce the complexity inherent in the representation of the twenty standard amino acids, we first used the same selection of amino acid grouping as the CT method. Then each protein is split into ten local regions of varying length and composition in order to better capture continuous and discontinuous PPI information from the sequence. For each local region, three local descriptors, composition (C), transition (T) and distribution (D), are calculated. C stands for the composition of each amino acid group along a local region. T represents the percentage frequency with which amino acid in one group is followed by amino acid in another group. D characterizes the distribution pattern along the entire region by measuring the location of the first, 25, 50, 75 and 100% of residues of a given group. For detailed descriptions of these descriptors, please refer to (Yang et al. 2010). Given that the amino acids are divided into seven groups in this instance, the calculation of these descriptors generates 63 attributes in each local region (7 for C, 21 for T and 35 for D). The descriptors for all local regions were combined, resulting in 630 features representing the general characteristics of the protein sequence. Thus, a 1260-dimensional vector has been built to represent each protein pair and used as a feature vector for input into SVM.

**Autocorrelation**

Autocorrelation features (Li et al. 2006) describe the level of correlation between two protein sequences in terms of their specific physicochemical property, which are defined based on the distribution of amino acid properties along the sequence. There are six amino acid properties used for deriving autocorrelation descriptors as the AC method. Here we use three commonly-used autocorrelation for predicting PPIs, i.e. Geary autocorrelation (GA) (Sokal and Thomson 2006), Moran autocorrelation (MA) (Horne 1988; Xia et al.2009), and Normalized Moreau-Broto autocorrelation (NA) (Feng and Zhang 2000). For the detailed descriptions of these autocorrelation descriptors, interested readers could refer to (Li et al. 2006).

**Performance measures**

Predicting a protein-protein pair as binding or non-binding is a binary classification problem, and many measures have been introduced for validation issues (Baldi et al. 2000). Here the prediction performances are evaluated by the overall prediction accuracy (ACC), sensitivity (SN), precision (PE) and Matthews correlation coefficient (MCC) (Matthews 1975) as follows:

$ACC=\frac{TP+TN}{TP+FP+TN+FN},$ $SN=\frac{TP}{TP+FN},$ $PE=\frac{TP}{TP+FP}$ （1）

and

$MCC=\frac{TP\times TN-FP\times FN}{\sqrt{(TP+FN)\times(TN+FP)\times(TP+FP)\times(TN+FN)}}$ （2）

where TP, TN, FP and FN denote true positive, true negative, false positive and false negative, respectively. In addition, we also used the receiver operating characteristic (ROC) curve (Zweig and Campbell 1993) to assess the prediction performance. An ROC curve is a [graphical](http://en.wikipedia.org/wiki/Graph_of_a_function) plot of the true positive rate (TPR) versus the false positive rate (FPR) for a [binary classifier](http://en.wikipedia.org/wiki/Binary_classifier) system as its discrimination threshold is varied. The TPR and FPR were defined as follows:

$TPR=\frac{TP}{TP+FN},$ $FPR=\frac{FP}{TN+FP}$ （3）

For each classifier, we tried different threshold values above which protein pairs were classified to interact, thereby obtaining a complete ROC curve. To summarize ROC curve in a single quantity, here the area under an ROC curve (AUC) is used. When the AUC value of a predictor is larger than the area of other ROC curves, the predictor is regarded as a better predictor.

**Table S1 Division of amino acids based on the dipoles and volumes of the** side chains

| No. | Group |
| --- | --- |
| 1 | A, G, V |
| 2 | C |
| 3 | D, E |
| 4 | F, I, L, P |
| 5 | H, N, Q, W |
| 6 | K, R |
| 7 | M, S, T, Y |

**Table S2 The original values of the six physicochemical properties for each amino a**cid

| Amino acid | H | VSC | P1 | P2 | SASA | NCISC |
| --- | --- | --- | --- | --- | --- | --- |
| A | 0.62 | 27.5 | 8.1 | 0.046 | 1.181 | 0.007187 |
| C | 0.29 | 44.6 | 5.5 | 0.128 | 1.461 | -0.03661 |
| D | -0.9 | 40 | 13 | 0.105 | 1.587 | -0.02382 |
| E | -0.74 | 62 | 12.3 | 0.151 | 1.862 | 0.006802 |
| F | 1.19 | 115.5 | 5.2 | 0.29 | 2.228 | 0.037552 |
| G | 0.48 | 0 | 9 | 0 | 0.881 | 0.179052 |
| H | -0.4 | 79 | 10.4 | 0.23 | 2.025 | -0.01069 |
| I | 1.38 | 93.5 | 5.2 | 0.186 | 1.81 | 0.021631 |
| K | -1.5 | 100 | 11.3 | 0.219 | 2.258 | 0.017708 |
| L | 1.06 | 93.5 | 4.9 | 0.186 | 1.931 | 0.051672 |
| M | 0.64 | 94.1 | 5.7 | 0.221 | 2.034 | 0.002683 |
| N | -0.78 | 58.7 | 11.6 | 0.134 | 1.655 | 0.005392 |
| P | 0.12 | 41.9 | 8 | 0.131 | 1.468 | 0.239531 |
| Q | -0.85 | 80.7 | 10.5 | 0.18 | 1.932 | 0.049211 |
| R | -2.53 | 105 | 10.5 | 0.291 | 2.56 | 0.043587 |
| S | -0.18 | 29.3 | 9.2 | 0.062 | 1.298 | 0.004627 |
| T | -0.05 | 51.3 | 8.6 | 0.108 | 1.525 | 0.003352 |
| V | 1.08 | 71.5 | 5.9 | 0.14 | 1.645 | 0.057004 |
| W | 0.81 | 145.5 | 5.4 | 0.409 | 2.663 | 0.037977 |
| Y | 0.26 | 117.3 | 6.2 | 0.298 | 2.368 | 0.023599 |

H, hydrophobicity; VSC, volume of side chains; P1, polarity; P2, polarizability;

SASA, solvent accessible surface area; NCISC, net charge index of side chains

**References**

Baldi P, Brunak S, Chauvin Y, Andersen CA, Nielsen H (2000) Assessing the accuracy of rediction algorithms for classification: an overview. Bioinformatics 16:412-424

Breiman L (1996) Bagging predictors. Mach Learn 24:123-140

Breiman L (2001) Random forests. Mach Learn 45:5-32

Feng Z, Zhang C (2000) Prediction of membrane protein types based on the hydrophobic index of amino acids. J Protein Chem 19:269-275

Guo Y, Yu L, Wen Z, Li M (2008) Using support vector machine combined with auto covariance to predict protein-protein interactions from protein sequences. Nucleic Acids Res 36:3025-3030

Horne D (1988) Prediction of protein helix content from an autocorrelation analysis of sequence hydrophobicities. Biopolymers 27:451-477

Li Z, Lin H, Han L, Jiang L, Chen X, Chen Y (2006) PROFEAT: a web server for computing structural and physicochemical features of proteins and peptides from amino acid sequence. Nucleic Acids Res 34:W32

Matthews B (1975) Comparison of the predicted and observed secondary structure of T4 phage lysozyme. Biochim Biophys Acta 405:442-451

Shen JW et al (2007) Predictina protein-protein interactions based only on sequences information. Proc Natl Acad Sci USA 104:4337-4341

Shi MG, Xia JF, Li XL, Huang DS (2010) Predicting protein-protein interactions from sequence using correlation coefficient and high-quality interaction dataset. Amino acids 38(3):891-899

Sokal R, Thomson B (2006) Population structure inferred by local spatial autocorrelation: an example from an Amerindian tribal population. American J Phys Anthropol 129:121-131

Xenarios I et al (2002) DIP, the Database of Interacting Proteins: a research tool for studying cellular networks of protein interactions. Nucleic Acids Res 30:303-305

Xia JF, Han, K, Huang, DS (2010) Sequence-based prediction of protein-protein interactions by means of rotation forest and autocorrelation descriptor. Protein Pept Lett 17(1):137-145

Yang L, Xia JF, Gui J, and Huang DS (2010) Prediction of protein–protein interactions from protein sequence using local descriptors. Protein Pept Lett. In press

Zweig M, Campbell G (1993) Receiver-operating characteristic (ROC) plots: a fundamental evaluation tool in clinical medicine. Clin Chem 39:561-577
